# Phytochemical Analysis, Anti-inflammatory, and Antioxidant Activities of *Dendropanax dentiger* Roots

**DOI:** 10.1155/2020/5084057

**Published:** 2020-11-20

**Authors:** Li Yang, Yiwei Fang, Ronghua Liu, Junwei He

**Affiliations:** ^1^Key Laboratory of Modern Preparation of TCM, Ministry of Education, Jiangxi University of Traditional Chinese Medicine, Nanchang 330004, China; ^2^First Affiliated Hospital of Gannan Medical University, Ganzhou 341000, China; ^3^Research Center of Natural Resources of Chinese Medicinal Materials and Ethnic Medicine, Jiangxi University of Traditional Chinese Medicine, Nanchang 330004, China

## Abstract

*Dendropanax dentiger* root is a traditional medicinal plant in China and used to treat inflammatory diseases for centuries, but its phytochemical profiling and biological functions are still unknown. Thus, a rapid, efficient, and precise method based on ultra high-performance liquid chromatography coupled with quadrupole time-of-flight tandem mass spectrometry (UHPLC-Q-TOF-MS/MS) was applied to rapidly analyse the phytochemical profiling of *D. dentiger* with anti-inflammatory and antioxidant activities *in vitro*. As a result, a total of 78 chemical compositions, including 15 phenylpropanoids, 15 alkaloids, 14 flavonoids, 14 fatty acids, 7 phenols, 4 steroids, 4 cyclic peptides, 3 terpenoids, and 2 others, were identified or tentatively characterized in the roots of *D*. *dentiger*. Moreover, alkaloid and cyclic peptide were reported from *D*. *dentiger* for the first time. In addition, the ethanol crude extract of D. dentiger roots exhibited remarkable anti-inflammatory activity against cyclooxygenase- (COX-) 2 inhibitory and antioxidant activities *in vitro*. This study is the first to explore the phytochemical analysis and COX-2 inhibitory activity of *D. dentiger*. This study can provide important phytochemical profiles and biological functions for the application of *D. dentiger* roots as a new source of natural COX-2 inhibitors and antioxidants in pharmaceutical industry.

## 1. Introduction

Over the past few years, secondary metabolites from natural products play an important role in the development of new drugs [1]. Higher plants represent sources of abundant phytochemicals with a wide range of biological effects and have attracted more attention in the past decades [2–6]. Consequently, most medicinal plants belong to higher plants have been widely to treat many human diseases in traditional folk medicine [1, 7–9]. Although numerous studies on the medicinal plants used as traditional Chinese medicines (TCMs), problems of chemical compositions and biological properties remained the main barriers in the development of modern traditional medicines or new drugs.

The genus *Dendropanax* (Araliaceae), known as “Shushen” in Chinese, comprises about 80 known species in tropical America and eastern Asia. In China, 16 native species have been found, which were widely cultivated in parks and/or used as folk medicine [10]. *D*. *dentiger* (Harms) Merr. is native to China and widely distributed in Guangxi, Jiangxi, Yunnan, and Guangdong provinces. In TCM, the roots of *D*. *dentiger* have been used as an important folk medicine for the treatment of inflammatory diseases [11]. Due to its potential pharmaceutical industry promoting effects, *D. dentiger* afforded structurally diverse and biologically active compounds, such as steroids, alkaloids, flavonoids, and monoterpenes; some of them showed potential anti-inflammatory, cytotoxic, and antioxidant activities [12, 13]. Although lots of chemical compositions report on *D. dentiger*, the full chemical profiling and COX-2 inhibitory activity of this plant have not yet been studied so far.

This study was the first time to determine the phytochemical profiling and COX-2 inhibitory activity. In addition, it was also to evaluate the antioxidant activity, including DPPH and ABTS assays *in vitro*. This finding may contribute to the processing and utility of *D*. *dentiger*.

## 2. Materials and Methods

### 2.1. Chemicals and Reagents

The COX-2 inhibitor screening assay kit was purchased from Beyotime Biotechnology (Shanghai, China). 2,2-Diphenyl-1-pircryhydrazyl (DPPH), 2,2′-azinobis-(3-ethylbenzthiazoline-6-sulphonate) (ABTS), and celecoxib were purchased from Sigma-Aldrich (St. Louis, MO, USA). L-Ascorbic acid (Vc) was purchased from Aladdin (Shanghai, China). Acetonitrile and formic acid (LC-MS grade) were purchased from Fisher Scientific (Pittsburgh, PA, USA). HPLC grade water was deionized using a Milli-Q ultrapure water system (Merck Millipore, Milford, MA, USA).

### 2.2. Plant Material

The roots of *D*. *dentiger* were collected in the town of Baidu, Baise City, Guangxi, China, in October 2016. A botanical voucher specimen of this plant (No. DD20161022) was deposited at authors' laboratory and was identified by one of the authors Ronghua Liu [10].

### 2.3. Extraction Procedure

The dried and powdered roots of *D*. *dentiger* (10.0 kg) were extracted with 95% EtOH (60L × 3) and subsequently 50% EtOH (60 L ×3) by maceration at room temperature for seven days. All filtrates were combined and evaporated under reduced pressure (EYELA, Tokyo, Japan) to obtain the ethanol crude extract of *D*. *dentiger* (DD, 1275 g, 12.75%).

### 2.4. UHPLC-Q-TOF-MS/MS

The UHPLC-Q-TOF-MS/MS was provided in our previously published article [14]. Chromatographic separation was conducted on a Luna Omega C18 (100 × 2.1mm, 1.6 *μ*m, Phenomenex Inc., CA, USA) keeping at 40°C. 0.1% aqueous formic acid (*v*/*v*, A) and acetonitrile (B) were used as mobile phases. The gradient elution with the flow rate of 0.3 mL/min was performed as follows: 0-15 min, 25% B; 15-18 min, 25%-55% B; 18-40 min, 55%-95% B; 40-42 min, 95% B for column cleaning, and a conditioning cycle time of 3 min with the same initial conditions of 5% B. The sample inject volume was 3 *μ*L.

### 2.5. COX-2 Inhibitory Assay

The anti-inflammatory effect of the sample against COX-2 inhibition was determined using colorimetric COX-2 inhibitor screening assay kit (no. S0168) and using celecoxib as the positive drug [2–4]. Briefly, 75 *μ*L of assay buffer, 5 *μ*L of cofactor working solution, and 5 *μ*L of working solution were mixed with 5 *μ*L of the sample at different concentrations and then incubated at 37°C. After 10 min, 5 *μ*L of probe and 5 *μ*L of substrate were added in all wells and then incubated at 37°C for 5 min, and the absorbance was determined (A_sample_). The absorbance of a blank (A_blank_) and control (A_control_) composed of only the sample and COX-2 enzyme solutions was also determined, respectively. The COX − 2inhibitoryactivity(%) = [(A_control_–A_sample_)/(A_control_–A_blank_)] × 100%.

### 2.6. Antioxidant Assay

#### 2.6.1. DPPH Radical Scavenging Activity

The DPPH radical scavenging activity of the sample was provided in our previously published articles [2–4]. Briefly, 150 *μ*L of DPPH solution (dissolved 0.2 mM in methanol) was mixed with 50 *μ*L of the sample at different concentrations. The mixture was stirred and incubated in the dark at 30°C for 30 min, and the absorbance was determined at 517 nm (A_sample_). The absorbance of a blank (A_blank_) and negative control (A_control_) composed of only the sample and DPPH solutions was also determined, respectively. The DPPH radical scavenging activity of the sample was calculated by the following equation: DPPHscavengingactivity = [1 − (A_sample_–A_blank_)/A_control_] × 100%. Vc was used as a positive control in this experiment.

#### 2.6.2. ABTS Radical Scavenging Activity

The ABTS radical scavenging activity of the sample was carried out using the method reported by Sun et al. with minor modification [15]. Briefly, 1.76 mL of K_2_S_2_O_8_ (140 mM) and 100 mL of ABTS solution (7 mM) were mixed and stored in the dark at 25°C for 12 h. Then, the ABTS stock solution was diluted with PBS (0.1 M, pH 7.4) until an absorbance value of 0.70 ± 0.02 was reached at 734 nm to obtain the diluted ABTS^+^ radical solution. Subsequently, 10 *μ*L of the sample was mixed with 195 *μ*L the diluted ABTS^+^ radical solution and incubated in the dark at 25°C for 106 min, and the absorbance of the sample at 734 nm (A_sample_) was measured. The absorbance of a blank (A_blank_) and negative control (A_control_) composed of only the sample and diluted ABTS^+^ radical solutions was also determined, respectively. The ABTS radical scavenging activity of the sample was calculated by the following equation: ABTSscavengingactivity = [1 − (A_sample_–A_blank_)/A_control_] × 100%. Vc was used as a positive control in this experiment.

### 2.7. Statistical Analysis

Graphpad Prism 6 was used for statistical analysis, and the data were presented as the means ± standarddeviation (SD). One-way analysis of variance (ANOVA) and Tukey's test were used for comparison of differences in groups. Differences with *p* < 0.05 indicated statistical significance.

## 3. Results and Discussion

### 3.1. Identification of Main Constituents in *D. dentiger* Root Extract

**Figure 1:**
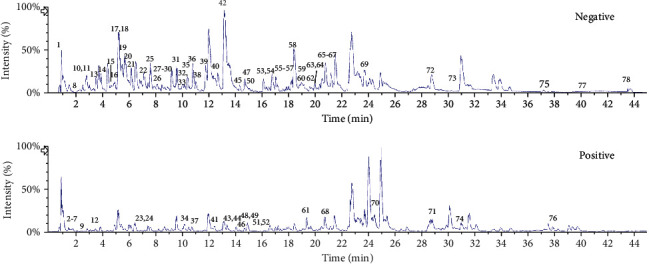
The base peak chromatograms of the *D*. *dentiger* root extract by UPLC-Q-TOF-MS/MS in negative and positive ion modes.

In the present study, the phytochemical compositions were identified using UHPL-Q-TOF-MS/MS based on the existing literatures and public databases, including ChemSpider, Massbank PubChem, and mzCloud, and summarized and described in Table 1 [16–44]. The base peak chromatograms of *D*. *dentiger* roots extract in positive and negative ion modes were presented in Figure 1. A total of 78 compounds, including 15 phenylpropanoids, 15 alkaloids, 14 flavonoids, 14 fatty acids, 7 phenols, 4 steroids, 4 cyclic peptides, 3 terpenoids, and 2 others, were identified. The molecular formula was accurately assigned within mass error of 5 ppm. Then, the fragment ions were used to further confirm the chemical structure. Furthermore, the fragmentation pathways of some representative compounds were proposed in order to facilitate structural identification. Among them, compounds 7, 10, 11, 13-32, 34-39, 41, 43-46, 48, 50-59, 61-65, 67-69, and 71-77 were reported for the first time in the Araliaceae family. Moreover, this is the first report on compounds 1-3, 5, 6, 9, 12, 40, 42, 47, 49, 60, 66, and 78 from the genus *Dendropanax* and compounds 4, 8, 33, and 70 from *Dendropanax dentiger* [12, 13].

#### 3.1.1. Phenylpropanoids

**Figure 2:**
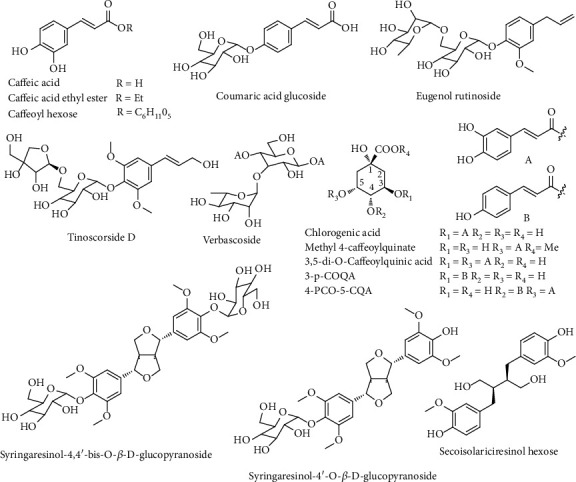
Chemical structures of phenylpropanoids from *D*. *dentiger* roots.

Phenylpropanoids were widely distributed in medicinal plants and its structures containing one or more C_6_-C_3_ units, which include three structure types, including simple phenylpropanoids, coumarins, and lignans [14]. A total of 15 phenylpropanoids in the roots of *D*. *dentiger* extract were identified in negative ion mode, including 12 simple phenylpropanoids and 3 lignans (Figure 2).

Compounds **17**-**19**, **21**, **22**, **26**, **27**, **36**, **38**, **42**, **50**, and **54** were simple phenylpropanoids, while compounds **27** and **36** were phenylpropanol and phenylpropene, respectively. Moreover, compounds **17**, **18**, **21**, **26**, **38**, **42**, **50**, and **54** were caffeic acid derivatives, including 4 caffeoylquinic acid derivatives (**18**, 2**6**, **42**, **50**). They combine by the quinic acid and caffeic acid with esteratic linkage and have similar cleavage pathways. The typical neutral losses of caffeoyl, quinine, H_2_O, and CO_2_ were the major cleavage pathway of such compounds. Taking compound **18** as an example, it gave the same MS^2^ base peak at *m/z* 191.0571 due to the loss of caffeic acid and a relatively intense secondary ion at *m/z* 179.0356, while the ion at *m/z* 161.0254 was produced by continuous loss of H_2_O, allowing the assignment of chlorogenic acid as reported by the reference data. The possible fragmentation mechanism was depicted in Figure S1. Besides, compound **42** also has the same fragmentation pathways.

Three compounds (**31**, **40**, **55**) belonging to the lignan, which contain two or more C_6_-C_3_ units. Compound **55** with a deprotonated molecule at *m/z* 523.21801 showed a base peak at *m/z* 361.1659 resulting from the loss of a hexosyl residue and was tentatively assigned as secoisolariciresinol hexose.

#### 3.1.2. Alkaloids

**Figure 3:**
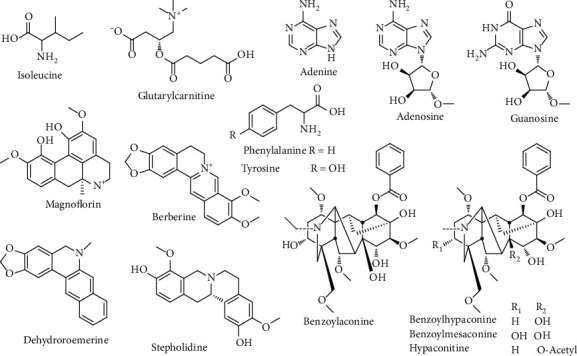
Chemical structures of alkaloids from *D*. *dentiger* roots.

In this study, a total of 15 alkaloids (Figure 3) in the roots of *D. dentiger* extract were identified, including 4 diterpenoid alkaloids (**44**, **48**, **52**, and **57**), 4 isoquinoline alkaloids (**23**, **24**, **37**, and **49**), 3 purine alkaloids (**2**, **4**, and **5**), 3 amino acid (**3**, **6**, and **9**), and 1 other alkaloid (**7**).

Compounds **44**, **48**, **52,** and **57** were diterpenoid alkaloids, which were belonging to aconitum alkaloids. In tandem mass spectrum of aconitum alkaloids commonly observe the neutral losses of H_2_O (18 Da), MeOH (32 Da), CO_2_ (44 Da), and PhCOOH (122 Da). Take the case of the **52**, it gave fragment ions at *m/z* 574.3045, 542.2772, 510.2458, and 105.0336 in the positive mode were corresponding to [M + H]^+^, [M + H–CH_3_OH]^+^, [M + H–2CH_3_OH]^+^, and [M + H–C_24_H_39_NO_8_]^+^, respectively. Compared with literature data, compound **52** was identified as benzoylhypaconine, and the possible fragmentation mechanism was depicted in Figure S2.

Compounds **23**, **24**, **37**, and **49** were isoquinoline alkaloids, which were widely distributed in medicinal plants and have high medicinal value. Compound **23** gave fragment ions at *m/z* 342.1718, 297.1133, 282.0899, and 265.0867 in the positive mode were corresponding to [M + H]^+^, [M + H–C_2_H_6_N]^+^, [M + H–C_2_H_6_N–CH_3_]^+^, and [M + H–C_2_H_6_N–CH_3_OH]^+^, respectively, of which ring B lost a C_2_H_6_N by a-cleavage and formed a Cp-ring; then, the ring A lost a methoxy at C-6 and formed an epoxy between C-6 and C-7. The tandem mass pattern of this compound was similar with magnoflorine. Thus, it could be identified as magnoflorine. The ESI–MS spectra of compound **24** exhibited similar quasi-molecular ions peak [M + H]^+^ at *m/z* 328.1557; their MS^2^ generated fragments at *m/z* 178.0862 and *m/z* 151.0759 by splitting of RDA on C-ring. Hence, compound **24** was tentatively identified as stepholidine. The [M + H]^+^ ion of compound **37** at *m/z* 278.1183 had a similar mass and fragmentation pathway to the dehydroroemerine, according to the characteristic ions at *m/z* 263.0948 [M + H–CH_3_]^+^, *m/z* 220.1129 [M + H–CH_2_O–CO]^+^, and *m/z* 204.0813 [M + H–CH_4_–CH_2_O–CO]^+^. For compound **49**, the positive mode MS spectrum showed the parent ion at *m/z* 336.1233 [M + H]^+^, and MS^2^ spectrum showed the fragment ions at *m/z* 321.1012 [M + H–CH_3_]^+^, 320.0937 [M + H–CH_4_]^+^, 306.0774 [M + H–2CH_3_]^+^, 292.0981 [M + H–CH_4_–CO]^+^, and 278.0822 [M + H–2CH_3_–CO]^+^. Compared with literature data, compound **49** was identified as berberine, and the possible fragmentation mechanism was depicted in Figure S3.

Moreover, other 7 alkaloid compounds **2**, **3**, **4**, **5**, **6**, **7**, and **9** were identified as adenine, tyrosine, adenosine, guanosine, isoleucine, glutarylcarnitine, and phenylalanine, respectively. To the best of our knowledge, alkaloid was reported from D. dentiger for the first time.

#### 3.1.3. Flavonoids

The mass spectra fragmentation patterns were widely used to provide the structural characterization of flavonoids in relation to the flavonoid aglycone and flavonoids glycoside. Moreover, the identification of the flavonoid aglycone was based on fragmentations, which related to the lost small neutral molecules and radicals (CH_3_, H_2_O, CO, and CO_2_), as well as the loss of a glucuronic acid (176 Da), hexose residue (162 Da), and apiose residue (132 Da) for flavonoids glycoside [14].

**Figure 4:**
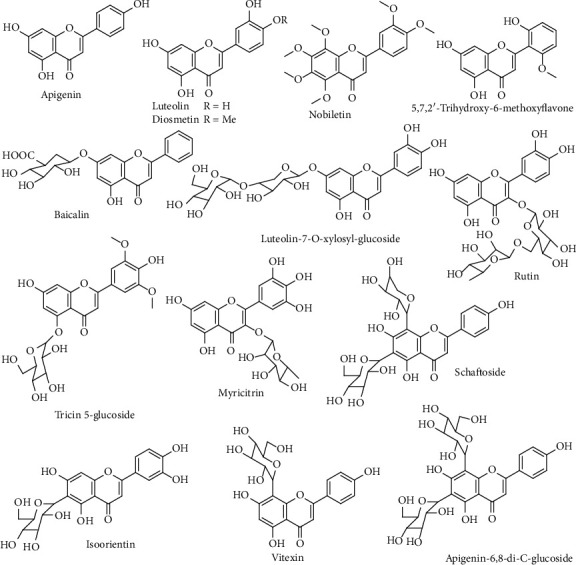
Chemical structures of flavonoids from *D*. *dentiger* roots.

In this study, 5 flavonoids (**39**, **53**, **56**, **61**, and **66**) and 9 flavonoid glycosides (**25**, **29**, **30**, **32**, **33**, **34**, **35**, **45**, and **47**) in the roots of *D*. *dentiger* extract were identified based on the molecular weight and fragmentation information (Figure 4). Compounds **39**, **53**, **56**, **61**, and **66** were belonging to flavonoids, which considered as 5,7,2′-trihydroxy-6-methoxyflavone, luteolin, diosmetin, nobiletin, and apigenin, respectively. The MS^2^ spectrum of **66** shown in Figure S4 was a representative example, which showed a [M–H]^–^ ion at *m/z* 269.0467, in accordance with the elemental composition of C_15_H_10_O_5_^–^.

Compounds **25**, **29**, **30**, **32**, **33**, **34**, **35**, **45**, and **47** were flavonoid glycosides, which considered as apigenin-6,8-di-C-glucoside, schaftoside, isoorientin, vitexin, rutin, myricitrin, luteolin-7-O-xylosyl-glucoside, tricin 5-glucoside, and baicalin, respectively. Compound **29** was C-glycosides, which the disaccharide substitution continuously loses 60, 90, and 120 Da fragment ions. Compound **29** showed a [M–H]^–^ ion at *m/z* 563.1414, C-6 substituted hexose broke up in ^0,4^X_0_, ^0,3^X_0_, and ^0,2^X_0_ to obtain 503.1196, 473.1092, and 443.0980 fragment ions, respectively, after that C-8 site pentose fractured at ^0,3^X1 and ^0,2^X_1_ to get 383.0781 and 353.0676, because the C-6 substituent glycosyl groups were superior to the C-8 replacement fracture. According to the characteristics of the fragment ions, compound **29** was easily confirmed as schaftoside. However, compound **33** was O-glycosides, which typically lost the entire sugar neutral molecule with significant loss of 132, 146, 162, and 192 Da fragments. Compound **33** [M−H]^−609.1490^ was rutin, which could be detected aglycone ion [Y_0_]^−301.0363^ and radical aglycone ion [Y_0_ − H]^−300.0284^ after losing carbohydrate continuously in the negative ion mode, and the possible fragmentation mechanism was depicted in Figure S5.

#### 3.1.4. Fatty Acids

In our study, a total of 14 fatty acids (peaks **16**, **58-59**, **62**, **65**, **67-69**, **71**, **72**, **74**, **75**, **77**, and **78**) were identified based on the reference mass spectra and databases.

#### 3.1.5. Phenols

A total of 7 phenols were identified in this study. Compounds **8**, **10**, **11**, **12**, **13**, **14**, and **28** were considered as gallic acid, 3-carboxy-4-hydroxy-phenoxy glucoside, vanillic acid hexose, syringic acid, methoxypolygoacetophenoside, piscidic acid, and syringaldehyde, respectively.

Take compound **11** as an example, its fragment ions at *m/z* 167.0359, 152.0128, 123.0474, and 108.0251 were identified as vanillic acid hexose. The ion at *m/z* 167.0359 was obtained by the loss of hexose, while the ion at *m/z* 123.0474 was produced by continuous loss of CO_2_. Meanwhile, the ion at *m/z* 152.0128 was obtained by the loss of CH_3_ from the precursor ion at *m/z* 167.0359, while the ion at *m/z* 108.0251 was produced by continuous loss of CO_2_. Based on the above fragment ions, which was obtained in the MS^2^ spectrum, the structure of compound **11** was easily confirmed as vanillic acid hexose.

#### 3.1.6. Steroids

Four steroids (peaks **63**, **64**, **73**, and **76**) were identified in this study. Peaks **63** and **64** generated [M + HCOO]^−^ ions at *m/z* 783.41695 and 1095.52283 in negative mode were unequivocally determined to be 25(27)-ene-timosaponin AIII and F-gitonin by comparison with the reference data. Peak **73** produced [M−H]^−^ ions at *m/z* 339.2323 in ESI^−^ mode. By comparing the quasi-molecular ions and fragmentations with MassBank and reference mass spectra data, peak **73** was tentatively identified as dimethisterone. Compound **76** had [M + H]^+^ ion at *m/z* 413.37809, and its fragments were at *m/z* 395.3701 [M + H–H_2_O]^+^, 255.2108 [M + H–C_10_H_20_–H_2_O]^+^, 213.1639 [M + H–C_10_H_20_–H_2_O–C_3_H_4_]^+^, 173.1328 [M + H–C_10_H_20_–H_2_O–2C_3_H_4_]^+^, and 159.1170 [M + H–C_10_H_20_–H_2_O–2C_3_H_4_–CH_2_]^+^, and was identified as *α*-spinasterol.

#### 3.1.7. Cyclic Peptides

A total of 4 cyclic peptides (peaks **41**, **43**, **46**, and **51**) were identified in this study, and the compounds **41**, **43**, **46**, and **51** showed [M + H]^+^ ion at *m/z* 679.51224, 792.59618, 905.68109, and 1018.76517, respectively. They have similar fragmentation pathways [14]. This is the first time to report the cyclic peptide from *D. dentiger*.

#### 3.1.8. Terpenoids

In current work, 3 terpenoids (peaks **15**, **20**, and **60**) were identified in negative ion mode.

Compound **15** had [M–H]^−^ ion at *m/z* 375.1307, and its fragments were at *m/z* 213.0777 [M–H–Glc]^–^, 169.0879 [M–H–Glc–CO_2_]^–^, and 151.0776 [M–H–Glc–CO_2_–H_2_O]^–^. Its fragmentation process was the same as the literature. Therefore, compound **15** was identified as mussaenosidic acid. Compound **20** exhibited a pseudomolecular ion at *m/z* 389.1098 [M–H]^−^ and fragment ions at *m/z* 345.1181 [M–H–CO_2_]^−^ corresponding to decarboxylation and *m/z* 165.0554 [M–H–CO_2_–C_6_H_12_O_6_]^−^ corresponding to the cleavage of elenolic acid moieties. Meanwhile, fragment ion at *m/z* 69.0405 was corresponding to the propiolic acid. Compound **20** was tentatively identified as oleoside. Compound **60** showed [M–H]^−^ ion at *m/z* 821.3970 and the fragment ions at *m/z* 351.0564 were corresponding to [2GluA–H_2_O]^−^, which the fragmentation pathways were similar with glycyrrhizin.

#### 3.1.9. Others

Compounds **1** and **70** were given [M + H]^+^ ions at *m/z* 341.10951 and 279.1589 and identified as sucrose and dibutyl phthalate, respectively, by comparing with literature.

### 3.2. COX-2 Inhibitory Assay

COX-2 is one of the most important proinflammatory enzyme of action for anti-inflammatory drugs, and celecoxib was a COX-2 selective inhibitor in clinical practice [2]. As observed in Table 2, the ethanol crude extract of *D. dentiger* roots showed significant COX-2 inhibitory effect with an IC_50_ value of 77.2 ± 4.2*μ*g/mL; however, there was an indicated remarkable difference (*p* < 0.01) in comparison with that of celecoxib with an IC_50_ value of 22.4 ± 1.4ng/mL. To the best of our knowledge, this study was the first time to determine the COX-2 inhibitory activity for *D. dentiger* [12, 13].

### 3.3. Antioxidant Activity

The DPPH and ABTS free radical scavenging activity assays were mostly used to evaluate the antioxidant effect of natural antioxidants [2]. Hence, the antioxidant activity of the *D. dentiger* roots ethanol crude extract was evaluated using ABTS and DPPH assays, and the results are shown in Table 2. The ethanol crude extract of *D. dentiger* roots showed the outstanding antioxidant activity, with IC_50_ values of 255.8 ± 10.3*μ*g/mL for DPPH assay and 151.9 ± 6.5*μ*g/mL for ABTS assay; however, there were exhibited significant differences (*p* < 0.01) comparable to those of the positive control V_c_ with IC_50_ values of 6.0 ± 0.2 and 1.2 ± 0.1*μ*g/mL, respectively.

To date, only one paper was reported the antioxidant activity of *D. dentiger*, and its ethyl acetate and n-butanol fractions showed significant against DPPH free radical scavenging activity [45]. Moreover, 7 phenolic compounds were isolated from the extract of *D. dentiger* and showed moderate or significant against DPPH free radical scavenging activity, with IC_50_ values of 0.038-0.741 *μ*M, comparable to that of V_c_ with an IC_50_ value of 0.059 *μ*M [45]. Therefore, this observed antioxidant activity could be due to the greater presence of secondary bioactive metabolites belonging to the flavonoids or phenolics noticed in ethanol crude extract of *D. dentiger* roots.

## 4. Conclusions

To summarize our findings, this study revealed that the root of *D. dentiger* was rich in phenylpropanoids, alkaloids, and flavonoids by UHPLC-Q-TOF-MS/MS and showed significant anti-inflammatory and antioxidant activities. This is the first study to describe the phytochemical profiling and COX-2 inhibitory activity of this plant [12, 13]. This study can provide important chemical information for the application of *D. dentiger* as a new source of natural COX-2 inhibitors and antioxidants in heath food and pharmaceutical industry.

## Figures and Tables

**Table 1:** Compounds identified from the roots of *D*. *dentiger* by UHPLC-Q-TOF-MS/MS in positive/negative ion mode.

No.	RT (min)	Ion mode	Molecular weight	Measured mass	Error (ppm)	Molecular formula	Fragments	Identification	Reference
Phenylpropanoids									
17	5.03	[M-H]^−^	342.09508	341.08806	0.7	C_15_H_18_O_9_	191.0568^a^, 179.0351, 173.0458, 135.0467, 93.0375	Caffeoyl hexose	16
18	5.23	[M-H]^−^	354.09508	353.08806	0.7	C_16_H_18_O_9_	353.0872, 191.0571^a^, 179.0356, 161.0254	Chlorogenic acid	16
19	5.59	[M-H]^−^	326.10017	325.09321	1	C_15_H_18_O_8_	176.0497, 163.0405, 145.0302, 119.0524^a^, 114.0389, 59.0167	Coumaric acid glucoside	17
21	5.91	[M-H]^−^	180.04226	179.03629	4.3	C_9_H_8_O_4_	135.0461^a^, 134.0386, 89.0430	Caffeic acid	18
22	6.89	[M-H]^−^	338.10017	337.0932	0.9	C_16_H_18_O_8_	191.0574^a^, 173.0460, 163.0410, 119.0529, 93.0385	3-p-COQA	19
26	7.93	[M-H]^−^	368.11073	367.10358	0.3	C_17_H_20_O_9_	193.0511, 191.0567^a^, 134.0389, 93.0377	Methyl 4-caffeoylquinate	19
27	8.12	[M-H]^−^	504.18429	503.17658	-0.9	C_22_H_32_O_13_	503.1761^a^, 341.1243, 221.0673, 161.0474, 101.0269	Tinoscorside D	20
31	9.56	[M-H]^−^	742.26842	741.26178	0.9	C_34_H_46_O_18_	417.1571^a^, 402.1310, 181.0524, 166.0289	Syringaresinol-4,4′-bis-*O*-*β*-D-glucopyranoside	21
36	10.77	[M-H]^−^	472.19446	471.18683	-0.8	C_22_H_32_O_11_	189.0565, 163.0773, 134.0381, 105.0375^a^, 89.0250, 71.0169	Eugenol rutinoside	22
38	11.00	[M-H]^−^	624.20542	623.19799	-0.3	C_29_H_36_O_15_	623.1974^a^, 461.1662, 179.0354, 161.0254^a^, 135.0459	Verbascoside	23
40	12.49	[M-H]^−^	580.21559	579.20843	0.2	C_28_H_36_O_13_	417.1550, 402.1323, 387.1019, 181.0516^a^, 166.0279, 151.0047	Syringaresinol-4′-*O*-*β*-D-glucopyranoside	21
42	13.18	[M-H]^−^	516.12678	515.11912	-0.7	C_25_H_24_O_12_	353.0878, 191.0566, 179.0359, 173.0466^a^, 161.0255, 135.0467	3,5-di-*O*-Caffeoylquinic acid	24
50	15.03	[M-H]^−^	500.13186	499.12418	-0.8	C_25_H_24_O_11_	353.0878, 337.0925, 191.0569, 179.0361, 173.0467^a^, 135.0463	4-PCO-5-CQA	19
54	16.76	[M-H]^−^	208.07356	207.06732	5	C_11_H_12_O_4_	161.0258, 135.0466, 133.0313^a^	Caffeic acid ethyl ester	21
55	17.52	[M-H]^−^	524.22576	523.21801	-0.9	C_26_H_36_O_11_	361.1659^a^, 346.1430, 317.1404, 231.0668, 161.0257	Secoisolariciresinol hexose	16
Alkaloids									
2	1.51	[M + H]^+^	135.0545	136.06184	0.5	C_5_H_5_N_5_	136.0627, 119.0356^a^, 107.0489, 92.0260, 91.0554^a^, 65.0414	Adenine	18
3	1.54	[M + H]^+^	181.07389	182.08102	-0.8	C_9_H_11_NO_3_	119.0492, 91.0561^a^, 77.0414, 65.0423	Tyrosine	25
4	1.59	[M + H]^+^	267.09675	268.10379	-0.9	C_10_H_13_N_5_O_4_	136.0622^a^, 119.0360	Adenosine	18
5	1.65	[M + H]^+^	283.09167	284.09882	-0.5	C_10_H_13_N_5_O_5_	152.0569^a^, 135.0308, 110.0364	Guanosine	25
6	1.71	[M + H]^+^	131.09463	132.10211	1.5	C_6_H_13_NO_2_	86.0987^a^, 72.9415, 69.0718, 57.0693, 55.9383, 55.0230	Isoleucine	25
7	1.72	[M + H]^+^	275.13689	276.14402	-0.5	C_12_H_21_NO_6_	276.1431, 258.1327, 230.1383^a^, 212.1256, 87.0330	Glutarylcarnitine	26
9	2.55	[M + H]^+^	165.07898	166.08619	-0.4	C_9_H_11_NO_2_	120.0811, 13.0550, 77.0412^a^	Phenylalanine	27
23	7.16	[M + H]^+^	341.16271	342.17031	1	C_20_H_23_NO_4_	342.1718, 297.1133, 282.0899, 265.0867^a^, 222.0899, 58.0696	Magnoflorine	26
24	7.31	[M + H]^+^	327.14706	328.15459	0.8	C_19_H_21_NO_4_	328.1557, 178.0862^a^, 163,0627, 151,0759	Stepholidine	28
37	10.85	[M + H]^+^	277.11028	278.11756	0	C_18_H_15_NO_2_	278.1183, 263.0948, 220.1129, 204.0813^a^	Dehydroroemerine	27
44	13.79	[M + H]^+^	589.2887	590.29653	0.9	C_31_H_43_NO_10_	590.2971^a^, 572.2870, 558.2670, 540.2608, 508.2340	Benzoylmesaconine	26
48	14.94	[M + H]^+^	603.30435	604.31125	-0.6	C_32_H_45_NO_10_	604.3141^a^, 554.2761, 242.1194	Benzoylaconine	26
49	15.00	[M + H]^+^	335.11576	336.1233	0.8	C_20_H_17_NO_4_	336.1244, 321.1012, 320.0937^a^, 306.0774, 292.0981, 278.0822	Berberine	26
52	15.83	[M + H]^+^	573.29378	574.30155	0.9	C_31_H_43_NO_9_	574.3045^a^, 542.2772, 510.2458, 105.0336	Benzoylhypaconine	26
57	17.95	[M + H]^+^	615.30435	616.31177	0.2	C_33_H_45_NO_10_	616.3162^a^, 556.2911, 524.2635, 338.1757, 161.0597	Hypaconitine	26
Flavonoids									
25	7.41	[M-H]^−^	594.15847	593.15094	-0.4	C_27_H_30_O_15_	593.1523, 503.1208, 473.1098, 383.0778, 353.0675^a^, 297.0772	Apigenin-6,8-di-C-glucoside	17
29	8.66	[M-H]^−^	564.14791	563.14072	0.2	C_26_H_28_O_14_	563.1414, 545.1304, 503.1196, 473.1092, 443.0980, 383.0781, 353.0676^a^, 297.0786, 173.0466, 93.0369	Schaftoside	19
30	8.91	[M-H]^−^	448.10056	447.09339	0.2	C_21_H_20_O_11_	357.0627, 339.0514, 327.0523^a^, 299.0569, 297.0410, 133.0307	Isoorientin	19
32	10.07	[M-H]^−^	432.10565	431.09862	0.6	C_21_H_20_O_10_	431.0991, 341.0666, 311.0567, 293.0454, 283.0616^a^, 161.0247, 117.0365	Vitexin	19
33	10.11	[M-H]^−^	610.15339	609.14652	0.7	C_27_H_30_O_16_	609.1490, 447.1153, 301.0363, 300.0284^a^, 271.0259, 255.0310, 161.0249	Rutin	19
34	10.15	[M + H]^+^	464.09548	465.10317	0.9	C_21_H_20_O_12_	303.0504^a^, 257.0444, 201.0546, 85.0309	Myricitrin	29
35	10.34	[M-H]^−^	580.14282	579.13562	0.1	C_26_H_28_O_15_	579.1365, 285.0411^a^	Luteolin-7-O-xylosyl-glucoside	19
39	11.76	[M-H]^−^	462.11621	461.10885	-0.2	C_22_H_22_O_11_	461.1063, 299.0560^a^, 284.0334, 256.0375	5,7,2′-Trihydroxy-6-methoxyflavone	30
45	14.18	[M-H]^−^	492.12678	491.11956	0.1	C_23_H_24_O_12_	491.1189^a^, 459.0923, 323.0771, 315.0732, 314.0442, 179.0361, 175.0398, 160.0178, 152.0134, 153.0208, 132.0219, 108.0286	Tricin 5-glucoside/tricin 7-glucoside	17
47	14.88	[M-H]^−^	446.08491	445.07734	-0.7	C_21_H_18_O_11_	269.0451^a^	Baicalin	30
53	16.06	[M-H]^−^	286.04774	285.04067	0.7	C_15_H_10_O_6_	285.0414, 257.0410, 243.0281,217.0511, 201.0205, 187.0389, 175.0420, 151.0053, 133.0311^a^, 132.0218, 105.0355, 83.0196, 65.0104	Luteolin	17
56	17.84	[M-H]^−^	300.06339	299.05669	1.9	C_16_H_12_O_6_	284.0313^a^, 256.0382, 227.0343, 151.0067	Diosmetin	19
61	19.12	[M + H]^+^	402.13147	403.13883	0.2	C_21_H_22_O_8_	403.1397, 388.1151, 373.0922^a^, 342.1110	Nobiletin	31
66	20.54	[M-H]^−^	270.05282	269.04576	0.8	C_15_H_10_O_5_	269.0467^a^, 241.0515, 225.0568, 213.0563, 197.0617, 181.0660, 171.0458	Apigenin	32
Fatty acids									
16	4.98	[M-H]^−^	176.06847	175.06192	4.1	C_7_H_12_O_5_	115.0411, 113.0637, 85.0689^a^	Hydroxy-methylglutaric acid	33
58	18.28	[M-H]^−^	228.13616	227.12906	0.8	C_12_H_20_O_4_	183.1407^a^, 165.1305	Dihydroxy dodecadienoic acid	34
59	18.40	[M-H]^−^	329.23349	329.2336	0.8	C_18_H_34_O_5_	329.2329, 229.1451, 211.1348, 209.1191, 171.1038^a^, 139.1141, 127.1144	Trihydroxy-octadecaenoic acid	35
62	19.55	[M-H]^−^	310.21441	309.20744	1	C_18_H_30_O_4_	209.1192^a^, 207.1408, 185.1193, 163.1139, 137.0985, 125.0991, 99.0849, 97.0682, 57.0403	Dihydroxy-octadecatrienoic acid	35
65	20.54	[M-H]^−^	314.24571	313.23872	0.9	C_18_H_34_O_4_	313.2389, 277.2178, 201.1139^a^, 199.0981, 171.1029, 127.1142, 125.0980	Dihydroxy-octadecaenoic acid	35
67	20.72	[M-H]^−^	312.23006	311.22326	1.5	C_18_H_32_O_4_	311.2234, 293.2129, 275.2005, 211.1353, 201.1141, 185.1188, 171.1040^a^, 139.1145, 127.1155	Dihydroxy-octadecadienoic acid	35
68	21.47	[M + H]^+^	352.26136	353.26862	-0.1	C_21_H_36_O_4_	261.2204, 187.1461, 145.1020, 131.0859, 107.0870, 93.0718, 81.0721^a^, 67.0580	Glyceryl linolenate	36
69	23.75	[M-H]^−^	294.2195	293.21295	2.5	C_18_H_30_O_3_	293.2114^a^, 249.2220, 197.1180, 185.1186, 125.0981, 113.0987	Hydroxy-octadecatrienoic acid	35
71	28.55	[M + H]^+^	354.27701	355.28463	1	C_21_H_38_O_4_	337.2750, 263.2377, 245.2258, 175.1478, 161.1327, 147.1159, 109.1017, 95.0862, 81.0720^a^	Glyceryl linoleate	36
72	29.08	[M-H]^−^	356.29266	355.28536	-0.1	C_21_H_40_O_4_	355.2758, 355.2918, 293.2837^a^, 295.0241, 240.9931	Monoolein	22
74	30.78	[M + H]^+^	330.27701	331.28412	-0.5	C_19_H_38_O_4_	313.2738, 109.1018, 95.0863, 85.1026, 81.0716, 71.0880, 57.0740^a^	Glycerin palmitate	36
75	37.15	[M-H]^−^	284.27153	283.2645	0.9	C_18_H_36_O_2_	283.2631^a^, 282.3573, 265.2527, 199.8494	Tearic acid or its isomer	35
77	40.40	[M-H]^−^	312.30283	311.29527	-0.9	C_20_H_40_O_2_	311.2948^a^, 311.1673, 293.2749, 184.0163, 183.0121	Arachidic acid/eicosanoic acid	37
78	43.36	[M-H]^−^	340.33413	339.32722	1.1	C_22_H_44_O_2_	339.1992, 184.0199, 183.0127^a^, 119.0516	Behenic acid	22
Phenols									
8	1.93	[M-H]^−^	170.02152	169.0152	4.6	C_7_H_6_O_5_	125.0254^a^, 124.0202, 97.0341, 79.0239, 69.0410	Gallic acid	38
10	2.80	[M-H]^−^	316.07943	315.07253	1.2	C_13_H_16_O_9_	153.0201, 152.0127, 108.0246^a^	3-Carboxy-4-hydroxy-phenoxy glucoside	19
11	2.94	[M-H]^−^	330.09508	329.08808	0.8	C_14_H_18_O_9_	167.0359, 152.0128^a^, 123.0474, 108.0251	Vanillic acid hexose	16
12	3.47	[M + H]^+^	198.05282	199.05998	-0.6	C_9_H_10_O_5_	181.0440, 140.0465, 125.0234^a^, 97.0297, 77.0406	Syringic acid	25
13	3.50	[M-H]^−^	360.10565	359.0987	0.9	C_15_H_20_O_10_	197.0465, 182.0234, 167.0000, 138.0344^a^, 123.0112, 95.0176	Methoxypolygoacetophenoside	33
14	3.82	[M-H]^−^	256.0583	255.05159	2.2	C_11_H_12_O_7_	225.0399, 196.0402,181.0514, 163.0405, 148.0190^a^, 135.0469, 120.0222, 109.0389, 95.0205	Piscidic acid	39
28	8.55	[M + H]^+^	182.05791	183.06503	-0.8	C_9_H_10_O_4_	140.0463, 125.0236, 95.0504, 77.0414^a^, 65.0421	Syringaldehyde	25
Steroids									
63	19.56	[M + HCOO]^−^	738.41904	783.41695	1	C_39_H_62_O_13_	783.4257, 737.4121^a^	25(27)-ene-Timosaponin AIII	35
64	19.91	[M + HCOO]^−^	1050.52469	1095.52283	0.9	C_50_H_82_O_23_	1049.5222^a^, 917.4771, 887.4673, 593.3684	F-Gitonin	35
73	30.23	[M-H]^−^	340.24023	339.23303	0.2	C_23_H_32_O_2_	339.2323, 163.1143^a^	Dimethisterone	35
76	38.29	[M + H]^+^	412.37052	413.37809	0.7	C_29_H_48_O	413.3786, 395.3701, 255.2108, 213.1639, 173.1328, 159.1170^a^, 145.1017, 109.0658	*α*-Spinasterol	40
Cyclic peptides									
41	12.63	[M + H]^+^	678.50438	679.51224	0.8	C_36_H_66_N_6_O_6_	679.5139^a^, 661.5044, 566.4337, 548.4185, 486.2149, 435.3332, 322.2482, 209.1660, 114.0932	Cyclo hexaleucyl (or isoleucyl)	35
43	13.71	[M + H]^+^	791.58845	792.59618	0.6	C_42_H_77_N_7_O_7_	792.5976^a^, 774.5873, 679.5137, 661.5069, 566.4308, 548.4199, 453.3648, 435.3336, 340.2605, 322.2510, 227.1767, 209.1654	Cyclo hetaleucyl (or isoleucyl)	35
46	14.58	[M + H]^+^	904.67251	905.68109	1.4	C_48_H_88_N_8_O_8_	905.6811^a^, 887.6715, 774.5847, 548.4196, 435.3353	Cyclo octaleucyl (or isoleucyl)	35
51	15.33	[M + H]^+^	1017.75658	1018.76517	1.3	C_54_H_99_N_9_O_9_	1018.7684^a^, 1000.7590, 887.6637	Cyclo nonaleucyl (or isoleucyl)	35
Terpenoids									
15	4.70	[M-H]^−^	376.13695	375.12984	0.5	C_16_H_24_O_10_	375.1307, 213.0777^a^, 169.0879, 151.0776, 113.0265	Mussaenosidic acid	18
20	5.79	[M-H]^−^	390.11621	389.10865	-0.7	C_16_H_22_O_11_	389.1098, 345.1181, 209.0459, 183.0691, 165.0554, 139.0781, 121.0672, 95.0526, 69.0405^a^	Oleoside	41
60	18.59	[M-H]^−^	822.40379	821.39586	-0.8	C_42_H_62_O_16_	821.3970^a^, 351.0564	Glycyrrhizin	42
Others									
1	0.90	[M-H]^−^	342.11621	341.10951	1.7	C_12_H_22_O_11_	179.0573, 161.0463, 119.0376, 113.0272, 89.0287, 71.0195, 59.0207^a^	Sucrose	43
70	24.37	[M + H]^+^	278.15181	279.1589	-0.7	C_16_H_22_O_4_	149.0233^a^, 121.0287	Dibutyl phthalate	44

^a^Base peak. RT: retention time; 3-p-COQA: 3-*O*-trans-coumaroylquinic acid; 4-PCO-5-CQA: 4-*O*-feruloyl-5-coumaroylquinic acid.

**Table 2:** IC_50_ values of *D*. *dentiger* root extract (DDR) and standards in COX-2, DPPH, and ABTS assays.

Samples	IC_50_ (*μ*g/mL)		
	COX-2 assay	DPPH assay	ABTS assay
DDR	77.2 ± 4.2^∗∗^	255.8 ± 10.3^∗∗^	151.9 ± 6.5^∗∗^
Celecoxib^a^	(22.4 ± 1.4) × 10^−3^	NT	NT
Vc^a^	NT	6.0 ± 0.2	1.2 ± 0.1

^a^Positive drug. NT: not tested. Data are shown as mean ± SD (*n* = 3). Differences were analyzed using ANOVA by Tukey's test. ^∗∗^*p* < 0.01 compared with the positive drug.

## Data Availability

The data used to support the findings of this study are available from the corresponding author upon request.

## Supplementary Materials

Supplementary Materials Fig. S1: tandem mass spectra and its fragmentation of chlorogenic acid in negative ion mode. Figure S2: tandem mass spectra and its fragmentation of benzoylhypaconine in positive ion mode. Figure S3: tandem mass spectra and its fragmentation of berberine in positive ion mode. Figure S4: tandem mass spectra and its fragmentation of apigenin in positive ion mode. Figure S5: tandem mass spectra and its fragmentation of rutin in positive ion mode.
