# Supplementary material for: Phytochemical Analysis, Anti-inflammatory, and Antioxidant Activities of Dendropanax dentiger Roots
Source: Biomed Res Int. 2020 Nov 20;2020:5084057. doi: 10.1155/2020/5084057 (PMC7700040; doi:10.1155/2020/5084057)
Supplement: Supplementary Materials — Fig. S1: tandem mass spectra and its fragmentation of chlorogenic acid in negative ion mode. Figure S2: tandem mass spectra and its fragmentation of benzoylhypaconine in positive ion mode. Figure S3: tandem mass spectra and its fragmentation of berberine in positive ion mode. Figure S4: tandem mass spectra and its fragmentation of apigenin in positive ion mode. Figure S5: tandem mass spectra and its fragmentation of rutin in positive ion mode. [file 5084057.f1.docx]

**Phytochemical analysis, anti-inflammatory and antioxidant activities of *Dendropanax dentiger* roots**

Li Yang ^1^, Yiwei Fang ^2^, Ronghua Liu ^1,*^, Junwei He ^3,*^

^1^ Key Laboratory of Modern Preparation of TCM, Ministry of Education, Jiangxi University of Traditional Chinese Medicine, Nanchang 330004, China

^2^ First Affiliated Hospital of Gannan Medical University, Ganzhou 341000, China

^3^ Research Center of Natural Resources of Chinese Medicinal Materials and Ethnic Medicine, Jiangxi University of Traditional Chinese Medicine, Nanchang 330004, China

*****Corresponding authors: rhliu@163.com (R.-H.L.); hjwjn2008@163.com (J.-W.H); Tel.: +86-791-87118873 (J.-W.H.)

**List of Supplementary data**

Fig. S1 Tandem mass spectra and its fragmentation of chlorogenic acid in negative ion mode…………………………………………………………………………………...3

Fig. S2 Tandem mass spectra and its fragmentation of benzoylhypaconine in positive ion mode……………………………………………………………………………….4

Fig. S3 Tandem mass spectra and its fragmentation of berberine in positive ion mode...5

Fig. S4 Tandem mass spectra and its fragmentation of apigenin in positive ion mode….6

Fig. S5 Tandem mass spectra and its fragmentation of rutin in positive ion mode……...7


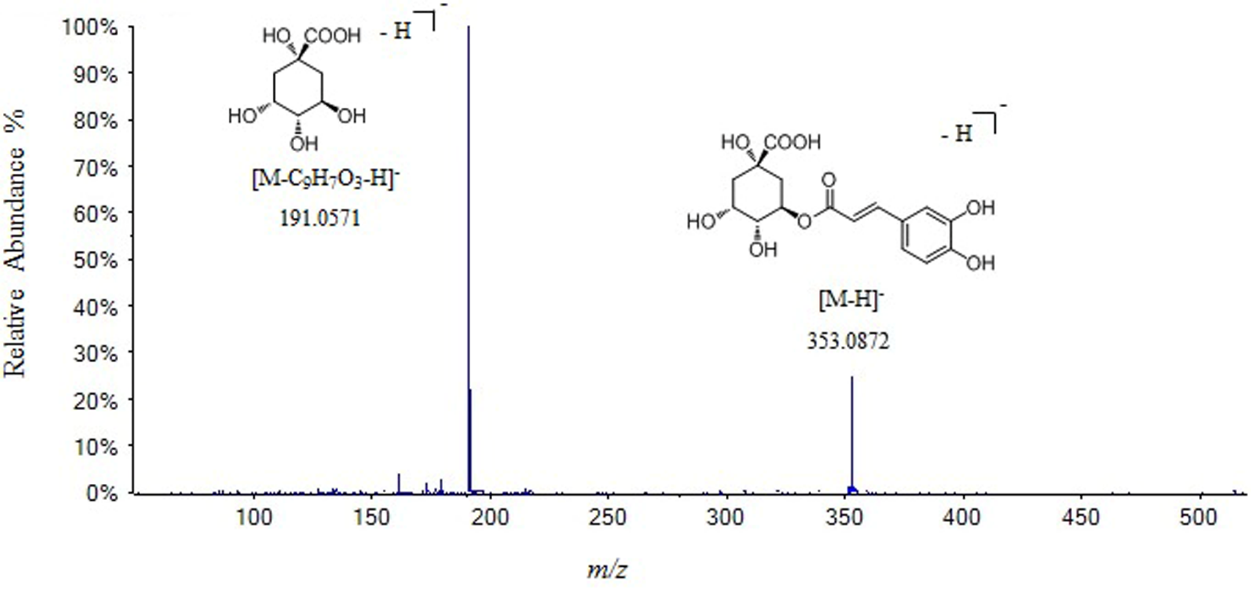


Fig. S1 Tandem mass spectra and its fragmentation of chlorogenic acid in negative ion mode.


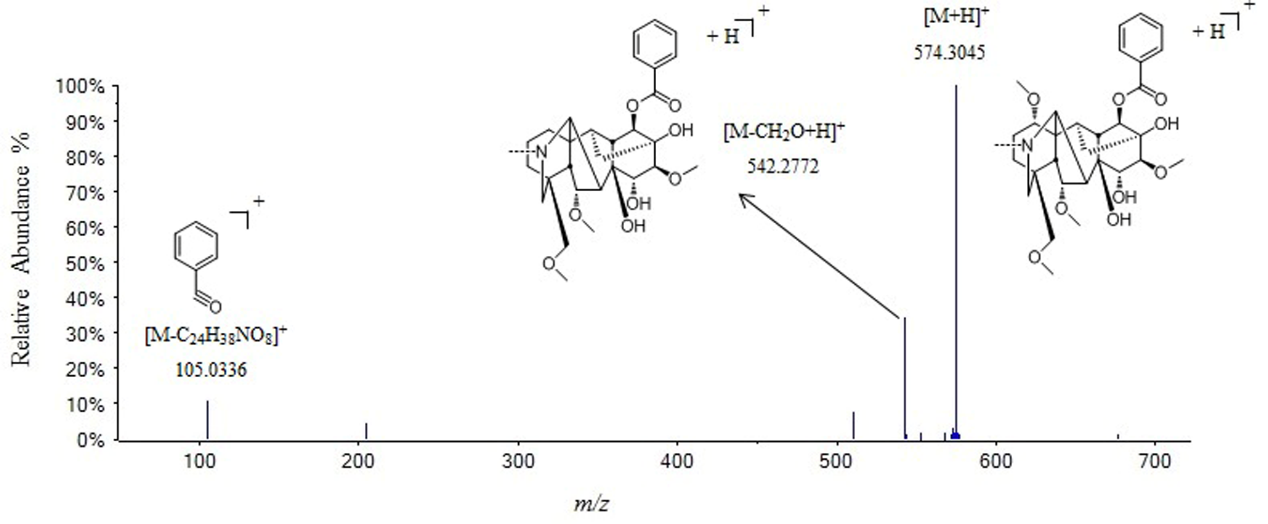


Fig. S2 Tandem mass spectra and its fragmentation of benzoylhypaconine in positive ion mode.


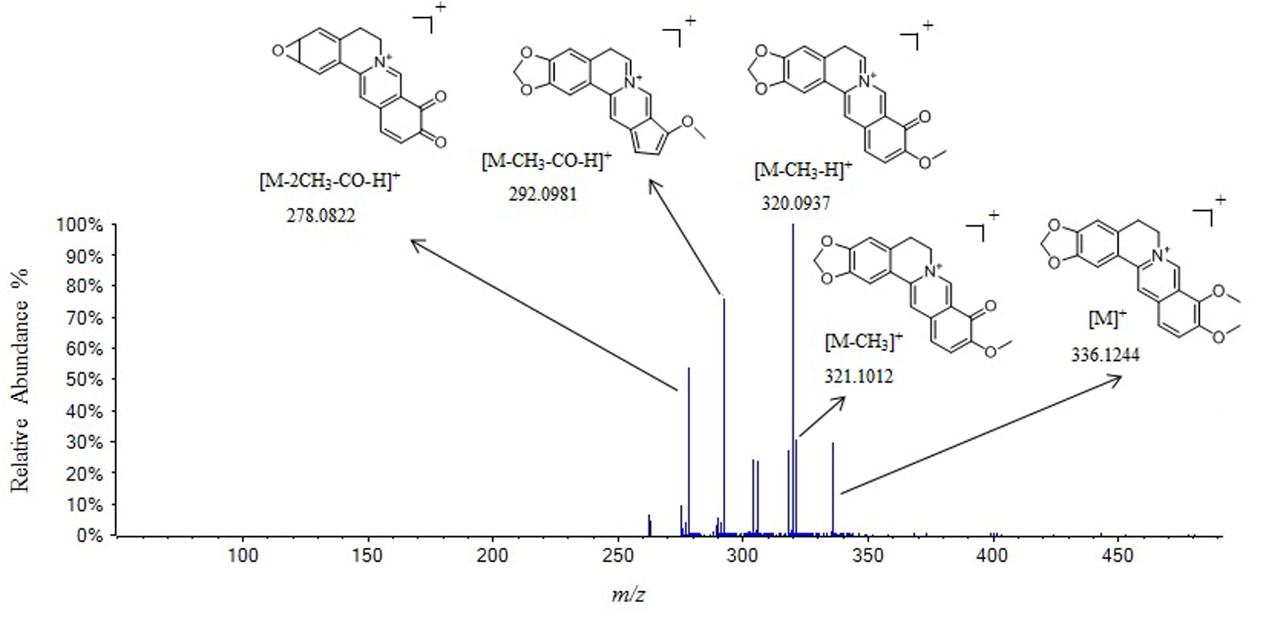


Fig. S3 Tandem mass spectra and its fragmentation of berberine in positive ion mode.


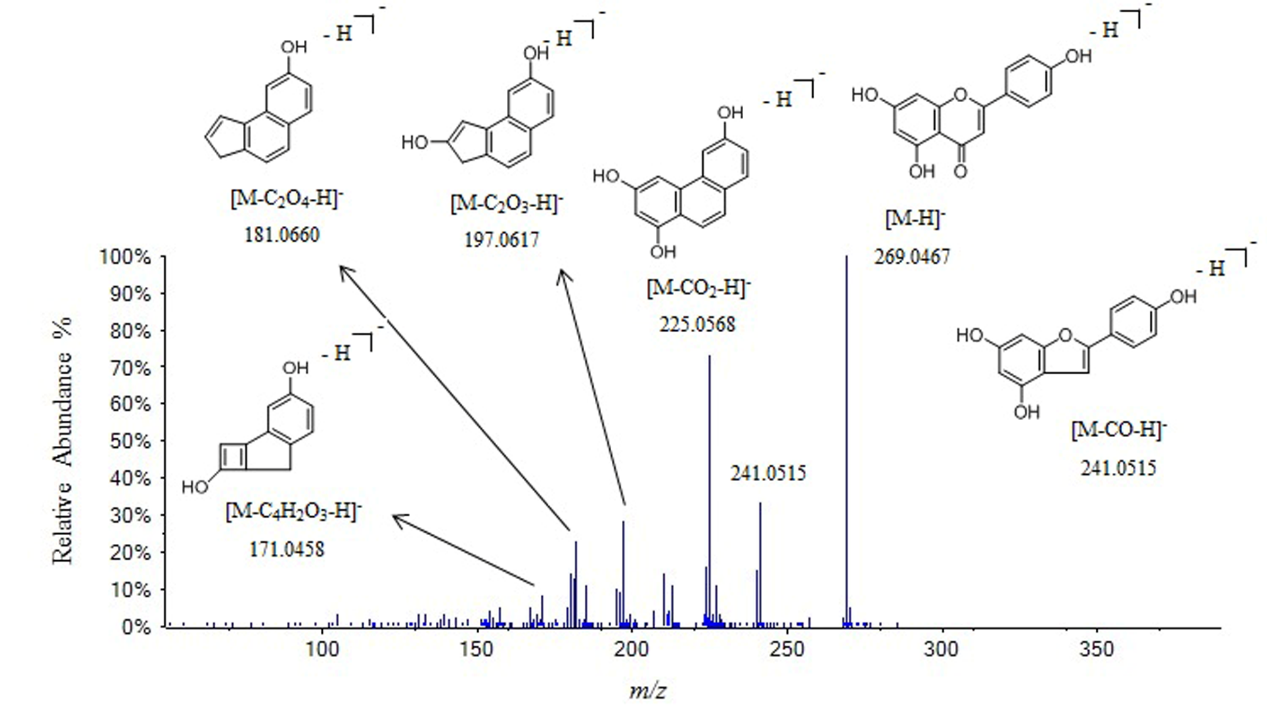


Fig. S4 Tandem mass spectra and its fragmentation of apigenin in positive ion mode.


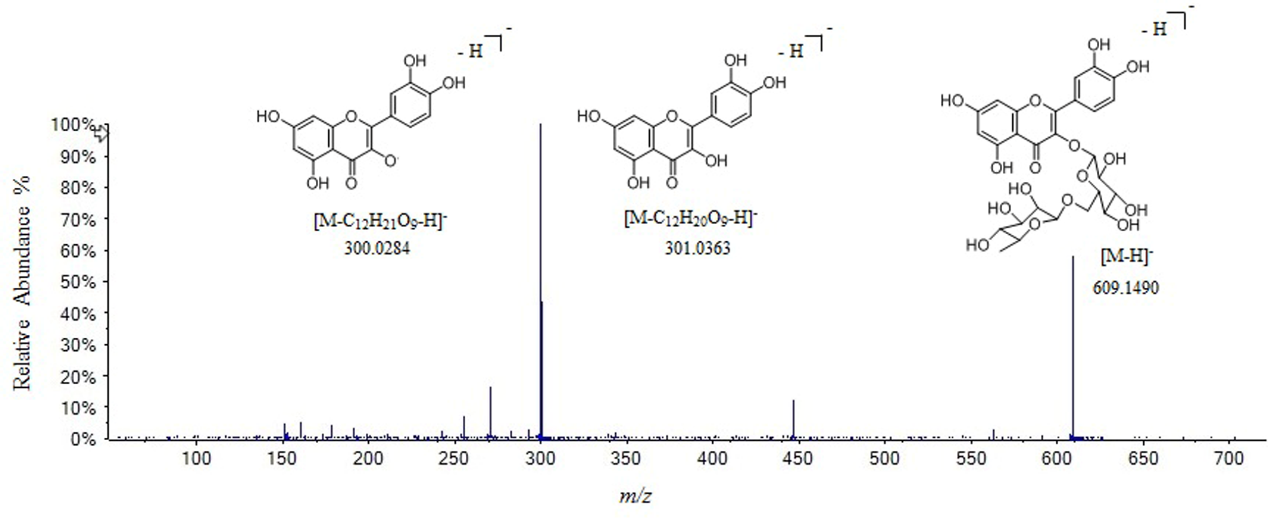


Fig. S5 Tandem mass spectra and its fragmentation of rutin in positive ion mode.
